# Developing a set of emergency department performance measures to evaluate delirium care quality for older adults: a modified e-Delphi study

**DOI:** 10.1186/s12873-024-00947-6

**Published:** 2024-02-15

**Authors:** Sarah Filiatreault, Sara A. Kreindler, Jeremy M. Grimshaw, Alecs Chochinov, Malcolm B. Doupe

**Affiliations:** 1 Department of Community Health Sciences, Rady Faculty of Health Sciences, University of Manitoba, 750 Bannatyne Ave, Winnipeg, MB R3E0W2 Canada; 2 Clinical Epidemiology Program, Ottawa Hospital Research Institute, Ottawa, ON K1H8L6 Canada; 3 Department of Medicine, University of Ottawa, Ottawa, ON K1H 8M5 Canada; 4 Department of Emergency Medicine, Rady Faculty of Health Sciences, University of Manitoba, 750 Bannatyne Ave, Winnipeg, MB R3E0W2 Canada

**Keywords:** Delphi technique, Performance measures, Emergency services (hospital), Delirium, Older adults, Process assessment (health care), Quality indicators (health care)

## Abstract

**Background:**

Older adults are at high risk of developing delirium in the emergency department (ED); however, it is under-recognized in routine clinical care. Lack of detection and treatment is associated with poor outcomes, such as mortality. Performance measures (PMs) are needed to identify variations in quality care to help guide improvement strategies. The purpose of this study is to gain consensus on a set of quality statements and PMs that can be used to evaluate delirium care quality for older ED patients.

**Methods:**

A 3-round modified e-Delphi study was conducted with ED clinical experts. In each round, participants rated quality statements according to the concepts of importance and actionability, then their associated PMs according to the concept of necessity (1–9 Likert scales), with the ability to comment on each. Consensus and stability were evaluated using a priori criteria using descriptive statistics. Qualitative data was examined to identify themes within and across quality statements and PMs, which went through a participant validation exercise in the final round.

**Results:**

Twenty-two experts participated, 95.5% were from west or central Canada. From 10 quality statements and 24 PMs, consensus was achieved for six quality statements and 22 PMs. Qualitative data supported justification for including three quality statements and one PM that achieved consensus slightly below a priori criteria. Three overarching themes emerged from the qualitative data related to quality statement actionability. Nine quality statements, nine structure PMs, and 14 process PMs are included in the final set, addressing four areas of delirium care: screening, diagnosis, risk reduction and management.

**Conclusion:**

Results provide a set of quality statements and PMs that are important, actionable, and necessary to a diverse group of clinical experts. To our knowledge, this is the first known study to develop a de novo set of guideline-based quality statements and PMs to evaluate the quality of delirium care older adults receive in the ED setting.

**Supplementary Information:**

The online version contains supplementary material available at 10.1186/s12873-024-00947-6.

## Introduction

Delirium is a serious condition of acute neurological dysfunction occurring in up to 38% of older emergency department (ED) patients [1–3]. Although older adults (i.e., people 65 years of age and older) are at high risk of developing delirium in the ED [4–7], it is missed in 57% to 85% of these patients [2, 8]. Lack of detection and treatment in the ED is associated with poorer outcomes such as increased length of hospital stay and mortality [7, 9–14]. Improving delirium care for older ED patients is hindered by underlying knowledge gaps and lack of practice standards for care in this setting [15]. Mechanisms to evaluate ED practice performance are needed to identify gaps and variations in quality care to focus delirium care improvement strategies where they are most needed.

Performance measures (PMs) are tools to quantify measurable aspects of practice performance [16, 17]. These are usually classified as structures (i.e., conditions under which care is provided) or processes (i.e., diagnosis, treatment, rehabilitation, and prevention of health conditions) of care as defined in Donabedian’s seminal framework of healthcare quality and measurement [18]. The extent that PMs are observed in practice provides an indication of the quality of the care provided and the likelihood of attaining optimal patient outcomes (i.e., changes in an individual or population attributable to healthcare) [17, 19, 20].

Numerous researchers and organizations assert that clinical practice guidelines (CPGs) are an essential first step in developing quality statements (i.e., concise statements defining best practice in a specific context), which in turn can be transformed into operationalizable metrics as PMs [16, 17, 19, 21–23]. Therefore, quality statements can be used to guide best practice and are an important antecedent to developing PMs that are necessary to evaluate the quality of care provided [16, 17, 19, 21]. In the past decade, work has been done to increase the methodological rigor of developing guideline-based PMs [16, 17, 24, 25]. Nothacker et al. (2016), as part of the Guidelines International Network (G-I-N), developed standards for generating guideline-based PMs [16], which have been used to inform our work.

Based on the results of an umbrella review of current delirium CPGs [26], we developed a preliminary set of quality statements and PMs grouped into four categories of delirium care: screening, diagnosis, risk reduction, and management. Criteria for quality statement and PM development from the umbrella review were: (1) agreement across two or more CPGs that the action or intervention be done, and (2) that the action or intervention was identified as a priority for implementation by at least one CPG group. No high-quality ED-specific delirium CPGs were found during our umbrella review, therefore we included CPGs from across the care continuum. However, the ED setting was included in the evidence base for many of the recommendations included in our synthesis [26]. To supplement the umbrella review and support the development of the PMs, structured searches of the Scopus and PubMed bibliographic databases were conducted for any recently published research relevant to delirium care for older adults in the ED specifically. Seven evidence syntheses [1–3, 27–30] and three multi-centre observational studies [8, 14, 31] were included as additional evidence to support the PMs.

Results from the umbrella review, supported by additional recent ED-specfic research, provided an evidence-based foundation for the creation of a set of quality statements (*N* = 10), and subsquent PMs (*N* = 24), for delirium care in the ED. Methods and criteria for conducting the transformation from CPG recommendation synthesis—to quality statement—to PM were incorporated into developing this preliminary set [16, 19, 25, 32]. For example, ensuring that each developed PM addresses an aspect of structure or process that is linked by evidence to improved outcomes, uses specific and unambiguous (i.e., concise) language, and is designed to be measurable [16, 19, 25, 32]. The greater number of PMs versus quality statements reflects the potential need to develop a structure and process PM from the same quality statement, or to develop more than one process PM to address the same quality statement to ensure the PMs are concise and are measurable. The next step in establishing a set of PMs for use was to conduct a formal consenus process with a diverse panel of experts to finalize a set of PMs from the transformed recommendations [16]. As the transformed recommendations were from CPGs for the entire care continuum, this next step was vitally important to ensure the final set of PMs are relevant to the ED, as well as to increase their credibility and acceptability in this setting [16, 23–25, 32–34].

The purpose of this study was to gain consensus on a set of guideline-based quality statements and PMs to guide and evaluate delirium care quality for older ED patients. To achieve this, we conducted a modified e-Delphi study to reach consensus among key clinical experts on a set of ED quality statements and PMs.

## Methods

**Fig. 1:**
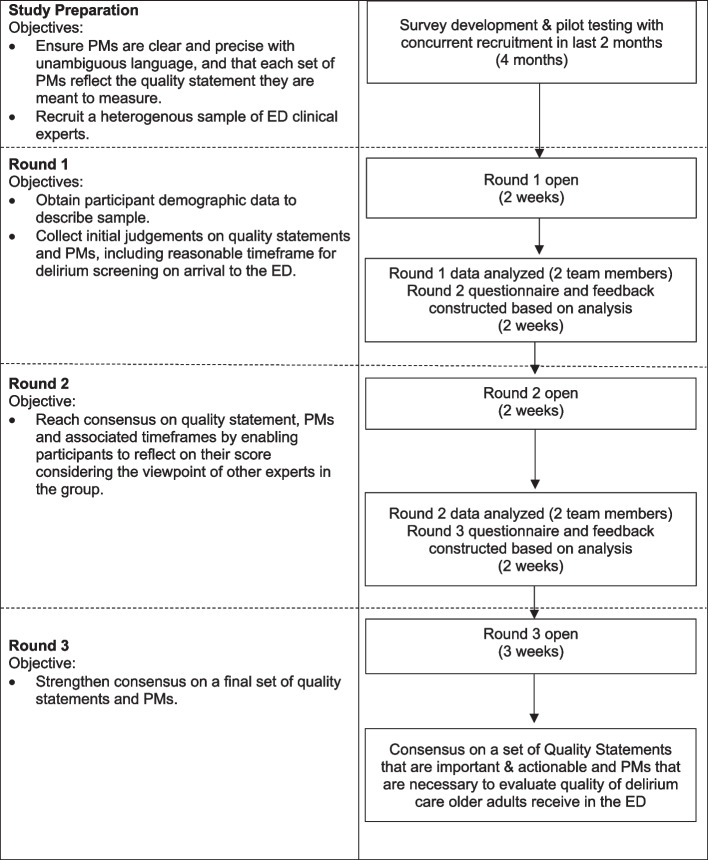
Delphi study flow

This 3-round modified e-Delphi study was conducted between April and July 2023. The methods of this study have been previously detailed in our open-access protocol [35], and are briefly described here. The design was informed by the Guidance on Conducting and REporting DElphi Studies (CREDES) [36], and other recommended criteria [33, 37]. This study received approval from the University of Manitoba Health Research Ethics Board (ID HS25728 [H2022:340]). Informed consent was received from all participants before completing any questionnaires. The consent process and each round was conducted electronically and anonymously using the Research Electronic Data Capture (REDCap) secure online platform for building and managing online surveys [38] through the University of Manitoba licensing agreement [39]. The study flow and objectives for each round are illustrated in Fig. 1.

### Study steering group

We convened a steering group with members consisting of co-authors (with methodological and clinical expertise), two patient/family representatives, and two further clinical experts (one ED physician and one registered nurse with experience as an ED front-line provider and clinical decision-maker). The steering group met at key stages to provide oversight on protocol design; to give feedback on Delphi questionnaire development, structure, and clarity; and to help identify potential Delphi participants. Including patient/family representatives in the steering group helped support the patient-centeredness of the quality statements and PMs by ensuring they contain aspects of care important to patients and families and suggesting alternative terminology that better reflects patient views. Members of the steering group who are not co-authors did not have access to raw study data and were not able to influence the study process. Feedback and changes suggested by the steering group were agreed between the study co-authors before implementation.

### Expert panel selection

An expert in this study is defined as one with clinical knowledge in the care of older adults in the ED.

Inclusion criteria:

- Able to read and write in English;
- Willing to participate, and;
- Meet one or more of the following criteria: (1) clinical experience in a relevant field to the ED care of older adults for five or more years post-basic graduation, (2) postgraduate qualifications or credentials relevant to the management of delirium in older adults, or (3) recognition by peers as an expert in the area (e.g., member of a relevant organization or network).

Exclusion criteria:

- Insufficient clinical knowledge and experience in a relevant area (e.g., system-level decision-maker, patient, or < 5 years post-basic clinical experience), or;
- Unable to commit to be available for the entire process.

### Recruitment

The initial recruitment period lasted 4 weeks to identify Canadian experts through existing professional networks and associations (e.g., Canadian Association of Emergency Physicians [CAEP] and National Emergency Nurses Association [NENA]); email invitations and advertisements; social media calls (LinkedIn, Twitter, and/or Facebook); and snowballing from other experts. Our minimum a priori sample size (*N* = 17) was not achieved after 4 weeks therefore, recruitment was extended for an additional 4 weeks and expanded to include eligible international clinical experts.

#### Survey design and development

The primary questionnaire in our study consisted of closed-ended questions with the opportunity to provide comments for each quality statement and related PMs to justify decisions and suggest edits to increase clarity. Each round was also accompanied by an introduction section to refamiliarize panelists to the study, state the intentions of the round, and provide definitions for key concepts.

Questionnaire development was informed by PM assessment instruments (i.e., AIRE [40] and QUALIFY [41]), criteria used by organizations that develop and implement PMs (i.e., the National Quality Forum [42] and NICE [19]), as well as syntheses of these sources [16, 32, 43]. Panelists were asked to judge each quality statement according to its importance and actionability; then, were asked to judge each related PM according to its necessity (see Table 1). The quality statements and PMs were scored using these criteria, on a Likert scale ranging from 1 to 9. Delphi participants were advised to think of each 9-point scale being made up of three parts (i.e., tertiles), where 1 to 3 could be used to record low ratings (i.e., not at all important, actionable and/or necessary), 4 to 6 record average ratings (i.e., somewhat important, actionable and/or necessary), and 7 to 9 record high ratings (i.e., very important, actionable and/or necessary). A response option of ‘I do not know’ was also provided to capture uncertainty. A question was included in the first two rounds on the Delphi to gain consensus on a reasonable timeframe to complete delirium screening upon arrival to the ED. This timeframe was incorporated in the associated quality statement and PM in the final round of the Delphi. A rationale for each quality statement and its PMs (including a summary of the evidence) was provided in each round to enable participants to make informed judgements.

**Table 1:** Selection criteria used to rate quality statements and PMs

Criteria	Definition
Important	Relevant and of crucial value to the care of older adults in the ED
Actionable	Care can be done by providers in the ED setting with appropriate resources and tools
Necessary	The PM is necessary to evaluate quality care for older adults in the ED (i.e., the quality statement)

Prior to implementing the Delphi survey, the questionnaire was piloted with clinical expert steering group members. The purpose of this process was to ensure that the PMs are clearly and precisely worded with unambiguous language, and that each set of PMs reflect the quality statement they are meant to measure. For example, as quality statements are defined as “concise statements defining best practice in a specific context” it was agreed to call the quality statements ‘best practices’ in the Delphi questionnaire to decrease the number of new concepts introduced to participants and to increase comprehensibility and readability of the survey. A copy of the Round 1 questionnaire that was approved for implementation by the steering group, which includes all preliminary ‘best practices’ (i.e., quality statements) and PMs, is provided in Supplemental File 1.

#### Procedure

In our modified e-Delphi process, a minimum of three rounds were planned a priori to allow participants to have feedback, revise previous responses, then stabilize responses [44, 45].

### Defining consensus and stability

We used the RAND criteria for agreement to define consensus [46], which aligns with PM development frameworks [32, 47]. Consensus was defined as 80% of ratings within the 3-point tertile of the overall median. The lower tertile (1–3) represents scores that are ‘not at all’, the middle tertile (4–6) represents scores that are ‘somewhat’, and the upper tertile (7–9) represents scores that are ‘very’ important, actionable, and/or necessary. To be included in the final set, quality statements and PMs needed to reach consensus in the upper tertile (i.e., overall panel median of 7 to 9, with 80% of ratings within the 3-point tertile of the overall median). Those that achieved consensus just below a priori thresholds were considered during qualitative data analysis and interpretation to determine justification for potential inclusion [37].

A measurement of stability was used as a stopping criterion for the Delphi process. This was defined as the consistency of responses between successive rounds (i.e., no meaningful change) [44, 45, 48]. Meaningful change was defined as a median change between tertiles *and* a greater than 15% change in the percentage of participants whose scores changed tertiles [45, 48].

### Stopping and PM removal criteria

For the overall study, the criterion to stop the Delphi process was defined as no meaningful change in scores between the current and preceding round on at least 75% of quality statements and PMs assessed. Additionally, criteria for PM removal were considered after the second round. To be removed from the process, a PM’s scores must have shown no meaningful change from the previous round, *and* there must have been consensus that the PM is not necessary (i.e., overall panel median of 1 to 3, with 80% of ratings in the lower tertile).

### Delphi rounds

In Round 1, along with the questionnaire containing the preliminary set of quality statements and PMs, participants also completed a participant demographics form. In Round 2, a personalized questionnaire was sent to each participant with: quantitative group results (i.e., median, minimum, and maximum ratings) presented numerically and graphically, qualitative feedback (i.e., summary of participants’ comments), and the participant’s own response to illustrate their position in relation to the group. In Round 3, a new personalized questionnaire was sent to each participant with the revised: quantitative group results presented numerically and graphically, qualitative feedback, and the participant’s own response to illustrate their position in relation to the group. Member checking of themes that emerged from feedback in Rounds 1 and 2 was also completed.

#### Data analysis

Descriptive statistics (frequency distributions) were calculated to determine if consensus, stability, PM removal and stopping criteria were met, as well as to present quantitative feedback to participants (median, minimum and maximum values) in rounds two and three. If a participant rated a question as ‘I do not know’ the value was counted as missing and the denominator was adjusted for that question to reflect the number of valid responses. Statistical analyses were performed using Microsoft Excel™ for Mac.

Inductive content analysis was used to code and summarize participants’ comments to be fed back to the group in rounds two and three, as well as to provide context during data interpretation and to support justification for including quality statements and PMs slightly below a priori quantitative thresholds in the final set [44, 49, 50]. Following a period of data familiarization, data were coded and counted iteratively to identify themes within each set of, as well as across all, quality statements and PMs. To be classified as a theme to be included in the comment summary for each quality statement and set of PMs, the code needed to be described by a minimum of two participants. Original wording from one expert that best represented the wording for that theme was used where possible [44, 50]. Across all rounds of the Delphi, overarching themes emerged from the coded data. To explore the trustworthiness of these results, extra questions were included in the final round of the Delphi as a form of participant validation (or member checking) [51].

## Results

### Delphi panel

Fifty-three experts were identified and contacted by the lead author, of which nine were known to snowball the invitation within their professional networks. Advertising by professional associations (e.g., CAEP, NENA, and iDelirium) through email fan-outs and/or social media reached approximately 8,000 individuals. Twenty-four experts expressed interest and met eligibility requirements. Of those, 22 provided consent and were enrolled into the study.

Over half of panelists were physicians (*n* = 12, 54.6%), followed by registered nurses (*n* = 6, 27.3%) (see Table 2). Over two-thirds of panelists’ primary work setting was the ED or urgent care (*n* = 15, 68.2%), and approximately two-thirds had 10 or more years of clinical experience (*n* = 14, 63.6%). Self-reported level of experience in older adult care on a 9-point Likert scale ranged from 5 to 9 (median = 7). Almost all panelists were from west or central Canada (*n* = 21, 95.5%). All panelists were retained in the final round of the Delphi (*N* = 22, 100%), with one missing response in Round 2.

**Table 2:** Demographic details of Delphi panel (*N* = 22)

Characteristics	Descriptive Statistics
Location^a^, *n* (%)	
British Columbia	3 (13.64%)
Alberta	6 (27.27%)
Saskatchewan	2 (9.09%)
Manitoba	8 (36.36%)
Ontario	2 (9.09%)
Europe	1 (4.55%)
Profession, *n* (%)	
Physician	12 (54.55%)
Registered Nurse	6 (27.27%)
Nurse Practitioner/Clinical Nurse Specialist	2 (9.09%)
Advanced Care Paramedic	2 (9.09%)
Highest level of education, *n* (%)	
Diploma	2 (9.09%)
Bachelor	4 (18.18%)
Postgraduate (MD)	8 (36.36%)
Master	6 (27.27%)
Doctorate (PhD)	2 (9.09%)
Primary work setting, *n* (%)	
Emergency Department/Urgent Care	15 (68.18%)
Acute Care	2 (9.09%)
University	1 (4.55%)
Critical Care Transport	4 (18.18%)
Years of post-basic experience, *n* (%)	
5 to 9	8 (36.36%)
10 to 14	3 (13.64%)
15 to 19	3 (13.64%)
20 to 24	1 (4.55%)
25 to 29	5 (22.73%)
30 or more	2 (9.09%)
Age in years, *n* (%)	
30 to 39	11 (50.00%)
40 to 49	1 (4.55%)
50 to 59	9 (40.90%)
60 to 69	1 (4.55%)
Gender^b^, *n* (%)	
Woman	14 (63.64%)
Man	8 (36.36%)
Self-reported level of experience in older adult care^c^	
Median (min to max)	7 (5 to 9)
Participant responses by Delphi round, *n* (%)	
Round 1	22 (100%)
Round 2	21 (95.45%)
Round 3	22 (100%)

^a^Zero participants from Quebec, Newfoundland, New Brunswick, Nova Scotia, Prince Edward Island, Yukon, Northwest Territories, or Nunavut

^b^Response options for non-binary (i.e., gender fluid, gender queer, agender, and non-binary), prefer not to answer, and prefer to specify further also available

^c^Likert scale 1 to 9 (1 indicating no experience [e.g., pediatric specialist] to 9 [e.g., completed special training in older adult care and/or many years specifically caring for older adults]

### Quality statement and PM selection

#### Quantitative results summary

Panelists evaluated 10 quality statements with 24 associated PMs, addressing four areas of delirium care (screening, diagnosis, risk reduction, and management). Criteria to stop the Delphi process were met after Round 3. Results were 98% stable between the second and third round. Quantitative results from all rounds are presented in Supplemental File 2. None of the PMs met removal criteria, therefore, all PMs and quality statements were retained in all three rounds to achieve or strengthen consensus on a final set. The experts reached consensus that six of the 10 quality statements were very important and very actionable (see Table 3). A further three quality statements were established as very important, but no consensus was reached as to their actionability; ratings ranged from 63.6% in the upper tertile for Quality Statement 08 (Multicomponent management) to 72.7% for Quality Statements 04 (Multicomponent risk reduction) and 09 (Cautious use of antipsychotic medications). For these nine quality statements, experts agreed that all associated PMs were very necessary, except for PM 06 (Repeat screening every shift) which fell below the a priori threshold (72.7%).

**Table 3:** Final quantitative quality statement and PM results (*N* = 22)

Quality Statement (QS) or PM	Round 3 Median Scores by tertile (%)		
	**Low**	**Mid**	**Upper**
**Screening**			
QS 01 (Screen for non-modifiable risk factors): Important	**8**		
	0.00	4.55	**95.45**
QS 01 (Screen for non-modifiable risk factors): Actionable	**8**		
	0.00	0.00	**100.00**
PM 01 (Evidence of structures to support screening): Necessary	**8**		
	0.00	4.55	**95.45**
PM02 (Older adults documented at risk): Necessary	**8**		
	0.00	18.18	**81.82**
PM03 (Non-modifiable risk factor screen): Necessary	**8**		
	0.00	9.09	**90.91**
QS 02 (Screen for delirium): Important	**9**		
	0.00	0.00	**100.00**
QS 02 (Screen for delirium): Actionable	**8**		
	0.00	9.09	**90.91**
PM 04 (Evidence of 4AT tool availability): Necessary	**8**		
	0.00	4.55	**95.45**
PM 05 (Screened on arrival): Necessary	**8**		
	0.00	13.64	**86.36**
PM 06 (Repeat screening every shift): Necessary	**9**		
	0.00	27.27	72.73
**Diagnosis**			
QS 03 (Assess and diagnose): Important	**9**		
	0.00	0.00	**100.00**
QS 03 (Assess and diagnose): Actionable	**9**		
	0.00	4.55	**95.45**
PM 07 (Diagnosis documented): Necessary	**9**		
	0.00	0.00	**100.00**
PM 08 (Diagnosis in discharge summary): Necessary	**8.5**		
	0.00	0.00	**100.00**
**Risk Reduction**			
QS 04 (Multicomponent risk reduction): Important	**9**		
	0.00	0.00	**100.00**
QS 04 (Multicomponent risk reduction): Actionable	**8**		
	4.55	22.73	72.73
PM 09 (Evidence of protocol/pathway): Necessary	**9**		
	0.00	0.00	**100.00**
PM 10 (Clinical risk factor assessment): Necessary	**8**		
	0.00	0.00	**100.00**
PM 11 (Risk reduction interventions): Necessary	**8**		
	0.00	4.55	**95.45**
QS 05 (Medication review): Important	**9**		
	0.00	0.00	**100.00**
QS 05 (Medication review): Actionable	**8**		
	0.00	4.55	**95.45**
PM 12 (Evidence of medication review tools): Necessary	**8**		
	0.00	4.55	**95.45**
PM 13 (Medication review completed): Necessary	**8**		
	0.00	0.00	**100.00**
QS 06 (Reduce unnecessary within-ED transfers): Important	**8**		
	4.55	4.55	**90.91**
QS 06 (Reduce unnecessary within-ED transfers): Actionable	4		
	31.82	40.91	27.27
PM 14 (Number of within-ED transfers): Necessary	6.5		
	9.09	40.91	50.50
**Management**			
QS 07 (Identify and treat causes): Important	**9**		
	0.00	0.00	**100.00**
QS 07 (Identify and treat causes): Actionable	**9**		
	0.00	4.55	**95.45**
PM 15 (Evidence of protocol/pathway): Necessary	**8**		
	0.00	4.55	**95.45**
PM 16 (Assess and identify causes): Necessary	**8**		
	0.00	0.00	**100.00**
QS 08 (Multicomponent management): Important	**9**		
	0.00	0.00	**100.00**
QS 08 (Multicomponent management): Actionable^a^	**7**		
	9.09	27.27	63.64
PM 17 (Evidence of protocol/pathway): Necessary	**8**		
	0.00	4.55	**95.45**
PM 18 (Management plan initiated): Necessary	**8**		
	0.00	0.00	**100.00**
PM 19 (Evidence of low stimulus care spaces): Necessary	**8**		
	0.00	4.55	**95.45**
PM 20 (Placed in low stimulus care space): Necessary	**8**		
	0.00	13.64	**86.36**
QS 09 (Cautious use of antipsychotic medications): Important	**9**		
	0.00	0.00	**100.0**
QS 09 (Cautious use of antipsychotic medications): Actionable	**7**		
	0.00	27.27	72.73
PM 21 (Evidence of risk of harm if antipsychotic given): Necessary	**8**		
	0.00	13.64	**86.36**
QS 10 (Communicate with patient and family/carers): Important	**9**		
	0.00	0.00	**100.00**
QS 10 (Communicate with patient and family/carers): Actionable	**8**		
	0.00	18.18	**81.82**
PM 22 (Evidence of communication tools): Necessary	**8.5**		
	0.00	0.00	**100.00**
PM 23 (Evidence of availability in other languages): Necessary	**8**		
	0.00	0.00	**100.00**
PM 24 (Patient and family/carers given information): Necessary	**8**		
	0.00	4.55	**95.45**

**Bolded numbers** = consensus criteria met

^a^Median tertile change and ≥ 15% change in responses between Round 2 and Round 3 (i.e., meaningful change)

Quality Statement 06 (Reduce unnecessary within-ED transfers) and its associated PM 14 did not meet criteria to be included in the final set. Although the Quality Statement reached consensus for importance (90.9% in upper tertile) only 27.3% rated it to be very actionable, and 50.0% rated the associated PM to be very necessary.

#### Qualitative results summary

Three overarching themes emerged from the qualitative responses, all related to the current actionability of the quality statements. The overall themes, organizing concepts for each theme presented to panelists in the final round, and examples of associated quotes (i.e., supporting quotes used to develop themes, as well as opposing theme quotes where applicable) are presented in Table 4. There was high agreement (95.5%) with first theme, ‘System-Level Impacts on the ED’, in which panelists described system-level issues, such as access block and ED crowding, thought to decrease the current actionability of the quality statements although they were perceived to be important. In contrast, panelists explained that the complex nature of bed management and patient flow in the ED made the reduction of within-ED transfers (Quality Statement 06) both non-actionable and unnecessary to evaluate. Instead of focusing on transfers within the ED, there was unanimous agreement with the second theme, ‘Prioritization for Transfer to Care Unit or Home’. Although panelists agreed older adults should be prioritized for transfer out of the ED, they also expressed the importance of improving care within the ED and shared ideas how some of the actionability issues could be addressed with adequate resources. These ideas are represented in the final theme, ‘Additional Healthcare Provider Supports’, which was also endorsed unanimously.

**Table 4:** Overarching themes, organizing concepts, and associated quote examples

Theme	Concept	Quote Examples
**System-Level Impacts on the ED**	The current state of the Canadian healthcare system, especially in EDs dealing with issues such as access block, crowding, staffing constraints, and competing priorities decreases the current actionability of many Quality Statements, although they may be important	**Supporting:** *“I believe this is extremely important however, due to staffing/time constraints, many of these are not possible to achieve”* *“I agree all very important interventions but the key would be with sufficient resources and plans on how to implement with staffing challenges *etc*.”* *“Again important but challenging to action without significant financial outlays and new direction by government and healthcare leadership.”* *“Current access block in the ED, staffing constraints, high acuity, competing priorities make this very difficult to take action on”* **Opposing:** *“… there will always be other competing priorities in the ED…. While we have to [be] practical in what an ED can achieve, we also need to realize that older adult care and delirium identification/management/prevention is a priority… It is critical we make it a priority, or else it will always be overlooked…”*
**Prioritization for Transfer to Care Unit or Home**	An important and valuable component of an ED care pathway/protocol for the risk reduction and management of delirium is to include prioritizing transfer of these patients to more appropriate care spaces (e.g., inpatient unit, geriatric observation unit, or home of usual residence with adequate supports)	**Supporting:** *“I think a more important priority is minimize ED length of stay for these patients, as prolonged time in the ED will invariably result in transfers with the department with our inability to control acuity and patient volumes”* *“Truly meaningful interventions would begin with early prevention. This should translate to early transfer out of ER [emergency room]”* *“I believe that patients would benefit from earlier transfer out of the ED to areas where these interventions are more easily implemented. However we should be striving to do better in the ED until this transfer is able to take place…”* **Opposing:** N/A
**Additional Healthcare Provider Supports**	It would be beneficial to have other practitioners and staff in the ED to support aspects of care, including: (1) Nurse Practitioners and/or Geriatric Emergency Management (GEM) nurses to aid in screening, assessment, and management; (2) Pharmacists or pharmacy technicians to aid in medication reviews and communication with General Practitioners/community care; and (3) Healthcare aids to assist with non-pharmacological care such as feeding, mobilizing, and toileting	**Supporting:** *“I think having a dedicated resource person within a busy ED to make sure this is done would be helpful. Perhaps this is a role that a nurse practitioner could be utilized”* *“I believe that having a pharmacist as part of the team in the ED is essential to review medication lists…”* *“We now are trialling HCAs [healthcare aides] in ER to assist with ADLs [activities of daily living] *etc. *& I think with the extra set of hands to do “comfort rounds”, monitoring is invaluable. Without them, time constraints limit my ability to continuously monitor for deteriorating”* **Opposing:** N/A

Qualitative results provide justification for including three quality statements that achieved consensus slightly below a priori thresholds (Quality Statements 04, 08, and 09). Many panelists who rated quality statements lower for actionability, rated the associated PMs as very necessary in recognition that the evidence generated from their measurement has the potential to guide improvement efforts (e.g., *“This is extremely important and valuable to the care of the patient. For statistical purposes, it would be good to know what proportion of patients are receiving this, but my impression would be that this would be dismal”, “Unlikely to achieve, but it would be nice to have this data to drive change”*, and *“Data important to guide further change”* [Experts rating quality statements lower for actionability and higher for PM necessity]). Lastly, PM 06 (Repeat screening every shift) reached consensus slightly below our a priori inclusion threshold as some participants viewed ongoing delirium screening being out of the scope of ED care (e.g., *“…The fact that these poor patients stay hours if not days in an ED screams system problem. The fact that the ED teams will have to do daily screens for these patients should be the real issue”* [Expert rating PM 06 in mid tertile]). While other participants thought repeat screening was necessary, as older adults tend to spend a longer time in the ED (e.g., *“I wonder if the frequency of every 24 h is not enough to capture and evaluate ED care. Perhaps every nursing shift (so twice a day)…”* and *“I think delirium screening should be occurring once per shift (every 12 h), given how quickly it can develop in the ED”* [Experts rating PM 06 in high tertile]). Due to known long standing issues with increased lengths of stay in EDs across Canada [52], and globally [53, 54], as well as increased incidence of delirium associated with ED lengths of stay over 10 h [3], it was decided to retain this PM for preliminary testing under the recognition that as care improves, attainment increases, and variability decreases over time, de-implementation of some of the PMs, such as PM 06, may be warranted [16].

#### Final set of quality statements and PMs

The final quality statement and PM set consisted of nine quality statements and 23 PMs, including nine structure PMs and 14 process PMs (see Table 5). Wording for some of the quality statements and PMs were modified after Rounds 1 and 2 based on panelist feedback. For example, multiple panelists viewed conducting delirium screening re-assessment once per nursing shift (instead of daily) was more practical. Two additions were also made based on expert consensus. First, a time benchmark of initial screening to be completed within 4 h of arrival to the ED was added, with 85.7% of panelists agreeing on this as a reasonable timeframe after two rounds. Second, prioritizing transfer to more appropriate care spaces was added as part of multicomponent interventions for risk reduction and management (Quality Statements 04 and 08) based on 100% agreement during the participant validation exercise. This addition is also supported by a recent systematic review and meta-analysis in which Oliveira and colleagues found that the odds of developing delirium increased over two-fold in older adults with ED lengths of stay over 10 h (OR, 2.23; 95% CI, 1.13–4.41) [3].

**Table 5:** Final set of quality statements and PMs by category

Quality Statement	PM(s)
**Screening**	
**QS 01:** All older adults (≥ 65 years of age) presenting to the ED will be identified as high-risk for delirium and assessed for other non-modifiable risk factors, including: • Cognitive impairment (past or present)/ dementia • Current fragility fracture (e.g., hip fracture), limb disfunction, or geriatric trauma • Severe illness with (or at risk for) deterioration • Nursing home residence • Hearing impairment • History of stroke	**PM 01:** Evidence of local structures, such as a prompt, checkbox, or automatic flag in the ED (electronic) health record to identify people at high-risk of developing delirium (including older age); (yes/no). (Structure) **PM 02:** Proportion of older adults presenting to the ED documented as being at risk for delirium on arrival; (%). (Process) **PM 03:** Proportion of older ED patients with documented assessment for other delirium risk factors upon initial assessment; (%). (Process)
**QS 02:** Older adults presenting to the ED will be screened for delirium using the 4AT tool^c^ within 4 hours^b^ of arrival, and at least daily afterwards	**PM 04:** Evidence of the ready availability of the 4AT tool in the ED setting (e.g., tool embedded in ED health record); (yes/no) (Structure) **PM 05:** Proportion of older adults presenting to the ED with a documented delirium screening using the 4AT within 4 hours^b^ of arrival; (%) (Process) **PM 06:** Proportion of older ED patients with a documented 4AT screening at least once every per shift^a^; (%) (Process)
**Diagnosis**	
**QS 03:** Older ED patients who have a positive screen for delirium will have an assessment and diagnosis by a trained healthcare professional (which can be the same person completing the screening) and have the diagnosis clearly documented in their health record (and written in a discharge summary^a^ when applicable)	**PM 07:** Proportion of older ED patients with a positive screen for delirium who have a formal diagnosis of delirium (or alternative diagnosis/reason for positive screen when applicable^a^) clearly documented in their health record; (%). (Process) **PM 08:** Proportion of older ED patients who are diagnosed with delirium and discharged from the ED that have the diagnosis written in a discharge summary^a^; (%). (Process)
**Risk Reduction**	
**QS 04:** Older adults presenting to the ED will receive a range of tailored interventions to prevent delirium based on an assessment of clinical factors, including: • Orientation/reorientation • Providing pain management • Promoting sleep hygiene • Optimizing hydration and nutrition • Optimizing of oxygen saturation • Mobilizing as soon as possible • Addressing infection • Regulating bladder and bowel function while avoiding unnecessary catheterization • Providing visual/hearing aids as necessary (i.e., sensory optimization) • Prioritizing transfer to more appropriate care spaces^b^	**PM 09:** Evidence of a readily available delirium protocol or care pathway for older ED patients to facilitate an assessment^a^ for clinical risk factors and tailor appropriate interventions to reduce the risk of delirium; (yes/no). (Structure) **PM 10:** Proportion of older ED patients who are assessed for clinical risk factors or delirium; (%). (Process) **PM 11:** Proportion of older ED patients who receive a range of tailored interventions (based on a clinical risk factor assessment) to reduce the risk of delirium; (%). (Process)
**QS 05:** Older ED patients will have a medication review completed by an experienced healthcare professional	**PM 12:** Evidence a readily available tool to aid in the review and identification of medications that may increase the risk of delirium (e.g., BEERS criteria or STOPP/START criteria); (yes/no). (Structure) **PM 13:** Proportion of older ED patients who have a medication review completed and documented by an experienced healthcare professional; (%). (Process)
**Management**	
**QS 07:** Older ED patients diagnosed^a^ with delirium will have a systematic assessment to identify and treat possible causes of delirium: • First, consider acute, life-threatening causes of delirium, including: low oxygen levels, low blood pressure, low glucose level, and drug/alcohol intoxication or withdrawal; • Second, identify other potential causes (e.g., medications, acute illness) noting multiple causes are common; • Third, optimize physiology and manage concurrent conditions	**PM 15:** Evidence of a readily available delirium care pathway in the ED to facilitate a systematic assessment; (yes/no). (Structure) **PM 16:** Proportion of older ED patients diagnosed^a^ with delirium who have a systematic assessment to identify causes of delirium; (%). (Process)
**QS 08:** Older ED patients with delirium will have a multicomponent management plan initiated while in the ED, including: • Cognitive engagement and reorientation • Promoting mobilization • Reviewing and adjusting medications • Promoting sleep hygiene • Providing visual and hearing aids (as necessary) • Regulating bladder and bowel function • Avoiding unnecessary stimuli (e.g., placing patient in care space with reduced noise) • Prioritizing transfer to more appropriate care spaces^b^	**PM 17:** Evidence of a readily available delirium care pathway in the ED to facilitate a multicomponent management plan; (yes/no). (Structure) **PM 18:** Proportion of older ED patients with delirium who have a multicomponent management plan initiated^a^ for the treatment of delirium; (%). (Process) **PM 19:** Evidence of local structures available within the ED for older adults with delirium to be placed in a care space with decreased unnecessary stimuli; (yes/no). (Structure) **PM 20:** Proportion of older ED patients with delirium who are placed in a care space with decreased unnecessary stimuli; (%). (Process)
**QS 09:** Older ED patients with delirium who are distressed/agitated or are a risk to themselves or others are not given antipsychotic medication (e.g., haloperidol) unless de-escalation techniques^d^ are ineffective or inappropriate^e^	**PM 21:** Proportion of older ED patients with delirium who have been given an antipsychotic medication (e.g., haloperidol) who were documented as being a risk to themselves or others and it is also documented that de-escalation techniques were ineffective or inappropriate; (%). (Process)
**QS 10:** Older ED patients with delirium and their family members/caregivers will be given information that explains the condition that meets the needs (cultural, language, cognitive) of the person; and family members/caregivers will be encouraged to be present in the ED^a^ and involved in delirium care pre and post discharge^a^, e.g., aiding in cognitive engagement and reorientation of the patient	**PM 22:** Evidence of a readily available communication tools (e.g., information pamphlets) in the ED to provide older adults with delirium and their family members/caregivers information that explains the condition; (yes/no). (Structure) **PM 23:** Evidence of information available in English, French, and other languages suited to local demographics (e.g., Indigenous languages) using plain language (e.g., Grade 6 reading level); (yes/no). (Structure) **PM 24:** Proportion of older ED patients with delirium and their families/carers^a^ who are given information explaining the condition; (%). (Process)

^a^Modified based on panel feedback/expert opinion

^b^Added based on panel consensus

^c^The ‘4 A’s test’ (https://www.the4at.com/) is a tool developed for clinical use on first presentation and contains four items assessing alertness, abbreviated mental test 4 (i.e., orientation), attention, and acute change. It is the recommended screening tool to use in the ED because it is quick (< 2 min), requires no special training, and has high diagnostic accuracy [14, 27, 55, 56]

^d^e.g., distraction, reassurance and verbal de‐escalation

^e^Worded to indicate only use with caution in urgent situations

## Discussion

There have been few attempts to establish PMs to evaluate quality of care for older ED patients in relation to delirium. Existing PMs are reported to be of low methodological quality and predominately based on pre-existing metrics [57–60]. PMs are only as good as the evidence and methods used to develop them [32, 43]. Poorly developed PMs can lead to unintended consequences by providing misleading information to guide decision-making, policy development, and quality improvement efforts [43]. There is general agreement in the ED quality of care literature that there is a need to rigorously develop new evidence-based PMs instead of basing work on pre-existing metrics [53, 57, 61]. In our study we developed a set of delirium quality statements and PMs for the care of older adults in the ED setting by conducting a formal consensus process. To our knowledge, this is the first known research to develop a de novo set of guideline-based metrics on this topic.

A diverse group of clinical experts reached consensus on a set of quality statements that are important, and associated PMs that are necessary to evaluate the quality of delirium care older adults receive in the ED. All quality statements in the final set reached consensus at or slightly below the a priori criterion for actionability, with a large caveat that much of this care would only be actionable with appropriate tools and resources available. There was overwhelming agreement that the current state of healthcare systems, especially in which many EDs are dealing with access block (inpatient boarding), crowding, and staffing constraints decreases the actionability of much of the care in the delirium quality statements, although there is agreement this care is important. Similarly, Eagles and colleagues (2022) found that ED clinicians identified delirium as important but it was not prioritized in the care of older ED patients [62]. Perceived lack of time, competing priorities, and increased demand have been identified as key barriers to delirium assessment and management by Canadian ED clinicians in two recent qualitative studies [62, 63]. These barriers to high-quality care delivery in the ED have only worsened in recent months [64] as the global pandemic has come to an end [65]. System-wide staffing shortages, bed closures, and pent-up demand have exacerbated access block and put increased pressures on EDs across Canada [64, 66, 67], as well as in many other countries such as the United States [68], United Kingdom [69], and Australia [70]. The one participant who disagreed with this theme in our study pointed out that there will always be competing priorities in the ED, and it is critical to make delirium care for older adults a priority or it will always be overlooked.

Despite system-level issues, clinical experts agreed more can be done within the ED to support the actionability of the quality statements and improve quality care. Implementing roles for other providers such as Nurse Practitioners and Geriatric Emergency Management (GEM) nurses were perceived to have great potential benefit in the screening, assessment, and management of delirium in the ED. Previous research has demonstrated that advanced practice nurses have a key role in successfully implementing practice change and improving ED quality care for older adults [71, 72]. Beyond the introduction of additional healthcare providers, tools are also needed to support ED clinicians to provide high-quality care.

The quality statements generated by this study can be used to guide practice change and enhance standardized electronic documentation. For example, they can be used to develop and embed risk reduction and management protocols, as well as embed screening tools into an electronic documentation system. Recent studies have reported improved delirium assessment and diagnosis in the ED with similar initiatives [73–75]. Further, embedding such tools and protocols will support reliable data capture, which will facilitate the ability to use the developed PMs to monitor and evaluate patient care. The PMs are necessary to provide baseline data to guide improvement efforts where they are most needed. Metrics such as these have been identified as an important component of quality improvement efforts, not only to provide evidence to governments and administrators, but also to increase frontline-provider awareness, enhance staff education, and increase buy-in [29, 73–75]. Prior to implementation, the PMs will undergo preliminary testing to ensure they are feasible to use to evaluate ED quality care [16].

This study has some key limitations. Despite our efforts to recruit broadly across Canada and internationally we encountered difficulties. This, unto itself, may speak to the increased burnout being experienced by ED providers world-wide since the pandemic [76]. Similarly, while we attempted to recruit other types of healthcare professionals (e.g., clinical educators and managers) none of these experts were willing to participate in our study. As most of our panelists were from Canada, this may limit the generalizability of our results. ED researchers can use the final set of quality statements and PMs from this study to repeat a similar consensus-building process with a different group of clinical experts to validate or further contextualize our results for different countries. Further, situational and personal biases can influence differences in how panelist make judgements when using the Delphi method [44]. We attempted to constrain these biases by limiting time between rounds, providing detailed background information and clear definitions for all concepts, providing quantitative and qualitative personalized feedback, as well as clearly defining consensus and stopping criteria.

## Conclusion

Our results confirm that high-quality delirium care is an important focus in the ED, although the quality statements and PMs were based on evidence from across the care continuum. This is the first known study to develop a set of guideline-based quality statements and PMs to evaluate the quality of care older adults receive in the ED setting. Results will be used in future research to test the feasibility of using the PMs to evaluate delirium care quality and guide improvement efforts.

### Supplementary Information

**Additional file 1: Supplemental File 1.** Round 1 Delphi Questionnaire as Presented in REDCap.

**Additional file 2: Supplemental File 2.** Quality Statement & PM Scores by Delphi Round.

## Data Availability

The datasets generated and analysed during the current study are available from the corresponding author on reasonable request.

## Abbreviations

ED: Emergency department
PM: Performance measure
CPG: Clinical practice guideline
G-I-N: Guidelines International Network
CREDES: Guidance on Conducting and REporting DElphi Studies
REDCap: Research Electronic Data Capture
CAEP: Canadian Association of Emergency Physicians
NENA: National Emergency Nurses Association
NICE: National Institute for Health and Care Excellence
GEM: Geriatric Emergency Management
