# Supplementary material for: Developing a set of emergency department performance measures to evaluate delirium care quality for older adults: a modified e-Delphi study
Source: BMC Emerg Med. 2024 Feb 15;24:28. doi: 10.1186/s12873-024-00947-6 (PMC10868025; doi:10.1186/s12873-024-00947-6)
Supplement: Supplementary file 1 — Additional file 1: Supplemental File 1. Round 1 Delphi Questionnaire as Presented in REDCap. [file 12873_2024_947_MOESM1_ESM.pdf]

Supplemental File 1. Round 1 Delphi Questionnaire as Presented in REDCap

AAA

University of Manitoba | Rady Faculty of Health Sciences

Round One Survey

Introduction

Delirium often goes unrecognized and undertreated in routine clinical care, including in the emergency department (ED), and is associated with poorer patient outcomes. Mechanisms to evaluate practice performance are needed to help focus delirium care improvement strategies where they are most needed.

This research project aims to create a set of performance measures (PMs) to evaluate the quality of delirium care provided to older ED patients. A vital part of this process is gaining expert consensus on a potential set of Best Practices & PMs that are important to ED care. The final set of PMs from this process will be tested in future research to assess their ability to be used to evaluate ED care quality.

We have done two things to develop this survey.

First, we conducted a systematic review to identify agreed upon best practices in delirium care. These best practices are grouped into four categories: **screening, diagnosis, risk reduction, and management**. A brief rationale is provided for each Best Practice to help inform your judgements.

Second, we created a list of potential PMs intended to capture the measurable aspects of each best practice (**24** PMs reflecting **10** Best Practices overall).

Definitions

Older adults: People 65 years of age and older

Performance measure (PM): Quantitative measure that can be used as a guide to evaluate and improve the quality of patient care

Process PM: Evaluates actions related to patient care (i.e., provider controlled)

Structure PM: Evaluates conditions under which care is provided (i.e., unit, hospital or system controlled)

Important: Relevant and has crucial value to the care of older adults in the ED setting

Actionable: Care can be done by providers in the ED setting with appropriate resources and tools

Necessary: The PM is necessary to evaluate quality care for older adults in the ED (i.e., the best practice)

Instructions

The evidence for the Best Practices & PMs included the entire spectrum of acute care. Therefore, it is essential to gain your opinion on which Best Practices & PMs are relevant to care in the ED specifically.

For each Best Practice and related PM(s) we are asking you to:

1. Rate each Best Practice according to how **important and actionable*** it is in the ED;
2. Review all the potential PMs and rate EACH ONE according to **how necessary it is** to measure the best practice in the ED;
3. Provide a rationale for your best practice and PM ratings no matter what your score is (this information will be confidentially summarized in the next questionnaire to help people reach consensus).

The Best Practices are meant to reflect optimal care for the health system and practitioners to strive towards.

The definition of 'actionable' recognizes a system must have appropriate supports in place for clinicians to provide high-quality care. Therefore, we want to know if you think care can and should be done in the ED.* ***Please do not judge* *best practices on site-specific resource constraints (e.g., recommended tool not currently used) or based on which provider you think may be responsible.*

-PLEASE COMPLETE THIS QUESTIONNAIRE by APRIL 16th-

Next Page >>

AAA

University of Manitoba | Rady Faculty of Health Sciences

Round 1 Questionnaire

Category 1: Screening

Instructions Page

Definition of scoring for each item according to important, actionable, or necessary**:**

1 to 3 = Not at all

4 to 6 = Somewhat

7 to 9 = Very

I don't know (DK) = Only choose this option if you feel you're not qualified to provide a response

BEST PRACTICE #1:

All older adults presenting to the ED should be identified as high-risk for delirium and assessed for other non-modifiable risk factors*.

Rationale

- Older age is widely acknowledged as a major risk factor for developing delirium.
- It is important to screen for non-modifiable risk factors upon initial presentation, and these factors should be used to define high-risk populations to target delirium screening and risk reduction strategies.

***Note:** *There is agreement across the evidence that cognitive impairment, current limb disfunction, severe illness, nursing home residence, hearing impairment, and history of stroke are other significant risk factors for ED delirium.*

FIRST, INDICATE IF YOU THINK THE BEST PRACTICE IS IMPORTANT & ACTIONABLE.

	Not at all 1	2	3	4	5	6	7	8	Very 9	DK
**Important: Is this important to the care of older adults in the ED?** * must provide value	○	○	○	○	○	○	○	○	○	○ reset
**Actionable: Can this be done in the ED with appropriate resources and tools?** * must provide value	○	○	○	○	○	○	○	○	○	○ reset

Please Comment: Use this section to justify your decision (e.g., If you scored between 1-3, why you think the Best Practice is not important or actionable, or if you scored between 7-9 why you think it is very important or actionable).

Expand

SECOND, INDICATE IF YOU THINK EACH PERFORMANCE MEASURE (PM) IS NECESSARY.

Necessary: Is the PM necessary to evaluate quality care for older adults in the ED?

	Not at all 1	2	3	4	5	6	7	8	Very 9	DK
**1. Structure PM:** Evidence of local structures, such as a prompt, checkbox, or automatic flag in the ED (electronic) health record to identify people at high-risk of developing delirium (including older age).	○	○	○	○	○	○	○	○	○	○ reset
**2. Process PM:** Proportion of older adults presenting to the ED documented as being at risk for delirium on arrival.	○	○	○	○	○	○	○	○	○	○ reset
**3. Process PM:** Proportion of older ED patients with documented assessment for other delirium risk factors upon initial assessment.	○	○	○	○	○	○	○	○	○	○ reset

Please Comment:

Use this section to justify your decision (e.g., If you scored between 1-3, why you think a specific PM is not necessary) or if you think something may be missing to better reflect the best practice.

Expand

Category 1: Screening

BEST PRACTICE #2:

Older adults presenting to the ED should be screened for delirium using the 4AT tool upon arrival*, and at least daily afterwards.

**Extra question to reach majority (or consensus) on a reasonable time interval*

Rationale

- Delirium is under-recognized in routine clinical care, and lack of detection is associated with poorer outcomes.
- Delirium screening should be done at the earliest opportunity for anyone in a high-risk population, such as older adults.
- The '4A's' test (4AT) is a tool developed for clinical use on first presentation and contains four items assessing alertness, orientation, attention, and acute changes. It is the recommended screening tool to use in the ED because it is quick (< 2 minutes), requires no special training, and has high diagnostic accuracy (even when used by healthcare professionals without specific training).

What is a reasonable timeframe to complete the 4AT test on arrival to the ED?

- ○ < 1hour
- ○ within 2 hours
- ○ within 4 hours
- ○ within 8 hours
- ○ within 12 hours
- ○ within 24 hours

reset

FIRST, INDICATE IF YOU THINK THE BEST PRACTICE IS IMPORTANT & ACTIONABLE.

	Not at all 1	2	3	4	5	6	7	8	Very 9	DK
**Important: Is this important to the care of older adults in the ED?** * must provide value	○	○	○	○	○	○	○	○	○	○ reset
**Actionable: Can this be done in the ED with appropriate resources and tools?** * must provide value	○	○	○	○	○	○	○	○	○	○ reset

Please Comment:
Use this section to justify your decision (e.g., If you scored between 1-3, why you think the Best Practice is not important or actionable, or if you scored between 7-9 why you think it is very important or actionable).

Expand

SECOND, INDICATE IF YOU THINK EACH PERFORMANCE MEASURE (PM) IS NECESSARY.

Necessary: Is the PM necessary to evaluate quality care for older adults in the ED?

	Not at all 1	2	3	4	5	6	7	8	Very 9	DK
**4. Structure PM:** Evidence of the ready availability of the 4AT tool in the ED setting (e.g., tool embedded in ED health record).	○	○	○	○	○	○	○	○	○	○ reset
**5. Process PM:** Proportion of older adults presenting to the ED with a documented delirium screening using the 4AT within x hours of arrival.	○	○	○	○	○	○	○	○	○	○ reset
**6. Process PM:** Proportion of older ED patients with a documented 4AT screening at least once every 24 hours.	○	○	○	○	○	○	○	○	○	○ reset

Please Comment:
Use this section to justify your decision (e.g., If you scored between 1-3, why you think a specific PM is not necessary) or if you think something may be missing to better reflect the best practice.

Expand

Category 2: Diagnosis

Instructions Page

Definition of scoring for each item important, actionable, or necessary:

1 to 3 = Not at all

4 to 6 = Somewhat

7 to 9 = Very

I don't know (DK) = Only choose this option if you feel you're not qualified to provide a response

BEST PRACTICE #3:

Older adults presenting to the ED with a positive screen for delirium should have the diagnosis clearly documented in their health record (and written in a discharge letter when applicable).

Rationale

- In clinical practice, delirium detection and diagnosis is missed in 57 to 85% of ED patients.
- Lack of detection and documentation is not only associated with poorer outcomes but also contributes to the continued underestimation of the true incidence and prevalence of delirium.

FIRST, INDICATE IF YOU THINK THE BEST PRACTICE IS IMPORTANT & ACTIONABLE.

	Not at all 1	2	3	4	5	6	7	8	Very 9	DK
**Important: Is this important to the care of older adults in the ED?** * must provide value	○	○	○	○	○	○	○	○	○	○ reset
**Actionable: Can this be done in the ED with appropriate resources and tools?** * must provide value	○	○	○	○	○	○	○	○	○	○ reset

Please Comment:

Use this section to justify your decision (e.g., If you scored between 1-3, why you think the Best Practice is not important or actionable, or if you scored between 7-9 why you think it is very important or actionable).

Expand

SECOND, INDICATE IF YOU THINK EACH PERFORMANCE MEASURE (PM) IS NECESSARY.

Necessary: Is the PM necessary to evaluate quality care for older adults in the ED?

	Not at all 1	2	3	4	5	6	7	8	Very 9	DK
**7. Process PM:** Proportion of older ED patients with a positive screen for delirium that have a formal diagnosis clearly documented in their health record.	○	○	○	○	○	○	○	○	○	○ reset
**8. Process PM:** Proportion of older ED patients who are diagnosed with delirium and discharged from the ED that have a discharge letter written in their record stating the diagnosis.	○	○	○	○	○	○	○	○	○	○ reset

Please Comment:

Use this section to justify your decision (e.g., If you scored between 1-3, why you think a specific PM is not necessary) or if you think something may be missing to better reflect the best practice.

Expand

Category 3: Risk Reduction

Instructions Page

Definition of scoring for each item important, actionable, or necessary:

1 to 3 = Not at all

4 to 6 = Somewhat

7 to 9 = Very

I don't know (DK) = Only choose this option if you feel you're not qualified to provide a response

BEST PRACTICE #4:

Older ED patients should receive a range of tailored interventions* to prevent delirium based on an assessment of clinical factors.

Rationale

- There is agreement across the evidence that older adults should receive a tailored multicomponent intervention to reduce the risk of delirium.
- For example, in an ED-specific systematic review and meta-analysis, Lee et al.(2022) found initiating a multicomponent strategy for older adults in the ED significantly reduced the risk of developing delirium (pooled OR 0.46; 95% CI 0.31 - 0.68).

***Note:** *Interventions supported by the evidence include orientation/reorientation, pain management, sleep hygiene, hydration and nutrition, oxygen saturation monitoring, mobilization as soon as possible, addressing infection, regulating bladder and bowel function while avoiding unnecessary urinary catheterization (e.g., supporting regular toileting), and providing visual/hearing aids (as needed).*

FIRST, INDICATE IF YOU THINK THE BEST PRACTICE IS IMPORTANT & ACTIONABLE.

	Not at all 1	2	3	4	5	6	7	8	Very 9	DK
**Important: Is this important to the care of older adults in the ED?** * must provide value	○	○	○	○	○	○	○	○	○	○ reset
**Actionable: Can this be done in the ED with appropriate resources and tools?** * must provide value	○	○	○	○	○	○	○	○	○	○ reset

Please Comment:
Use this section to justify your decision (e.g., If you scored between 1-3, why you think the Best Practice is not important or actionable, or if you scored between 7-9 why you think it is very important or actionable).

Expand

SECOND, INDICATE IF YOU THINK EACH PERFORMANCE MEASURE (PM) IS NECESSARY.

Necessary: Is the PM necessary to evaluate quality care for older adults in the ED?

	Not at all 1	2	3	4	5	6	7	8	Very 9	DK
**9. Structure PM:** Evidence of a readily available delirium protocol or care pathway for older ED patients to assess for clinical risk factors and tailor appropriate interventions to reduce the risk of delirium.	○	○	○	○	○	○	○	○	○	○ reset
**10. Process PM:** Proportion of older ED patients who are assessed for clinical risk factors of delirium.	○	○	○	○	○	○	○	○	○	○ reset
**11. Process PM:** Proportion of older ED patients who receive a range of tailored interventions (based on a clinical assessment) to reduce the risk of delirium.	○	○	○	○	○	○	○	○	○	○ reset

Comments:
Use this section to justify your decision (e.g., If you scored between 1-3, why you think a specific PM is not necessary) or if you think something may be missing to better reflect the best practice.

Expand

Category 3: Risk Reduction

BEST PRACTICE #5:

Older ED patients should have a medication review completed by an experienced healthcare professional.

Rationale

- Exposure to certian high-risk medications (e.g., according to BEERS criteria) increases the odds of developing delirium.
- Conducting a medications review can decrease the incidence of delirium.

Note: *There is agreement across CPGs and evidence syntheses that there is insufficient or inconclusive evidence for the use of any medications to reduce the risk of delirium.*

FIRST, INDICATE IF YOU THINK THE BEST PRACTICE IS IMPORTANT & ACTIONABLE.

	Not at all 1	2	3	4	5	6	7	8	Very 9	DK
**Important: Is this important to the care of older adults in the ED?** * must provide value	○	○	○	○	○	○	○	○	○	○ reset
**Actionable: Can this be done in the ED with appropriate resources and tools?** * must provide value	○	○	○	○	○	○	○	○	○	○ reset

Please Comment:

Use this section to justify your decision (e.g., If you scored between 1-3, why you think the Best Practice is not important or actionable, or if you scored between 7-9 why you think it is very important or actionable).

Expand

SECOND, INDICATE IF YOU THINK EACH PERFORMANCE MEASURE (PM) IS NECESSARY.

Necessary: Is the PM necessary to evaluate quality care for older adults in the ED?

	Not at all 1	2	3	4	5	6	7	8	Very 9	DK
**12. Structure PM**: Evidence a readily available tool to aid in the review and identification of medications that may increase the risk of delirium (e.g., BEERS criteria or STOPP/START criteria).	○	○	○	○	○	○	○	○	○	○ reset
**13. Process PM**: Proportion of older ED patients who have a medication review completed and documented by an experienced healthcare professional.	○	○	○	○	○	○	○	○	○	○ reset

Please Comment:

Use this section to justify your decision (e.g., If you scored between 1-3, why you think a specific PM is not necessary) or if you think something may be missing to better reflect the best practice.

Expand

Category 3: Risk Reduction

BEST PRACTICE #6:

Unnecessary transfers or moves within the ED should be avoided for older ED patients.

Rationale

- Recommendations for avoiding unnecessary transfers are based on evidence for promoting orientation and reorientation (i.e., the more times a person is transferred within and between hospital units, the more disorientating it can become).
- In a recent Cochrane systematic review and network meta-analysis, Burton et al. (2021) found reorientation significantly reduces the risk of older people developing delirium (OR 0.32; 95% CI 0.11 - 0.89).

FIRST, INDICATE IF YOU THINK THE BEST PRACTICE IS IMPORTANT & ACTIONABLE.

	Not at all 1	2	3	4	5	6	7	8	Very 9	DK
**Important: Is this important to the care of older adults in the ED?** * must provide value	○	○	○	○	○	○	○	○	○	○ reset
**Actionable: Can this be done in the ED with appropriate resources and tools?** * must provide value	○	○	○	○	○	○	○	○	○	○ reset

Please Comment:
Use this section to justify your decision (e.g., If you scored between 1-3, why you think the Best Practice is not important or actionable, or if you scored between 7-9 why you think it is very important or actionable).

Expand

SECOND, INDICATE IF YOU THINK THE PERFORMANCE MEASURE (PM) IS NECESSARY.

Necessary: Is the PM necessary to evaluate quality care for older adults in the ED?

14. **Process PM**: Number of recorded transfers or moves an older ED patient has while in the ED.	Not at all 1	2	3	4	5	6	7	8	Very 9	DK
	○	○	○	○	○	○	○	○	○	○ reset

Please Comment:
Use this section to justify your decision (e.g., If you scored between 1-3, why you think a specific PM is not necessary) or if you think something may be missing to better reflect the best practice.

Expand

Category 4: Management

Instructions Page

Definition of scoring for each item important, actionable, or necessary:

1 to 3 = Not at all

4 to 6 = Somewhat

7 to 9 = Very

I don't know (DK) = Only choose this option if you feel you're not qualified to provide a response

BEST PRACTICE #7:

Older ED patients with a positive screen for delirium should have an assessment to identify and treat possible causes of delirium.

Rationale:

- The main focus of delirium management is identifying and treating the underlying cause (or combination of causes).
- Assessment, facilitated by the use of an established care pathway, should be conducted to manage delirium.

FIRST, INDICATE IF YOU THINK THE BEST PRACTICE IS IMPORTANT & ACTIONABLE.

	Not at all 1	2	3	4	5	6	7	8	Very 9	DK
**Important: Is this important to the care of older adults in the ED?** * must provide value	○	○	○	○	○	○	○	○	○	○ reset
**Actionable: Can this be done in the ED with appropriate resources and tools?** * must provide value	○	○	○	○	○	○	○	○	○	○ reset

Please Comment:
Use this section to justify your decision (e.g., If you scored between 1-3, why you think the Best Practice is not important or actionable, or if you scored between 7-9 why you think it is very important or actionable).

Expand

SECOND, INDICATE IF YOU THINK EACH PERFORMANCE MEASURE (PM) IS NECESSARY.

Necessary: Is the PM necessary to evaluate quality care for older adults in the ED?

	Not at all 1	2	3	4	5	6	7	8	Very 9	DK
**15. Structure PM:** Evidence of a readily available delirium care pathway in the ED for the documentation of an assessment.	○	○	○	○	○	○	○	○	○	○ reset
**16. Process PM:** Proportion of older ED patients with a positive delirium screen who have a documented assessment to identify causes of delirium.	○	○	○	○	○	○	○	○	○	○ reset

Please Comment:
Use this section to justify your decision (e.g., If you scored between 1-3, why you think a specific PM is not necessary) or if you think something may be missing to better reflect the best practice.

Expand

Category 4: Management

BEST PRACTICE #8:

Older ED patients with a positive screen for delirium should have a multicomponent* management plan initiated while in the ED.

Rationale

- There is agreement across all high-quality CPGs that delirium should be managed non-pharmacologically using a tailored multicomponent intervention or care pathway.

***Note:** *Components supported by the evidence include cognitive engagement and reorientation, mobilization, medication review, sleep hygiene, visual/hearing aids (as needed), regulating bladder and bowel function, and avoiding unnecessary stimuli (e.g., placing patient in care space with reduced noise).*

FIRST, INDICATE IF YOU THINK THE BEST PRACTICE IS IMPORTANT & ACTIONABLE.

	Not at all 1	2	3	4	5	6	7	8	Very 9	DK
**Important: Is this important to the care of older adults in the ED?** * must provide value	○	○	○	○	○	○	○	○	○	○ reset
**Actionable: Can this be done in the ED with appropriate resources and tools?** * must provide value	○	○	○	○	○	○	○	○	○	○ reset

Please Comment:
Use this section to justify your decision (e.g., If you scored between 1-3, why you think the Best Practice is not important or actionable, or if you scored between 7-9 why you think it is very important or actionable).

Expand

SECOND, INDICATE IF YOU THINK EACH PERFORMANCE MEASURE (PM) IS NECESSARY.

Necessary: Is the PM necessary to evaluate quality care for older adults in the ED?

	Not at all 1	2	3	4	5	6	7	8	Very 9	DK
**17. Structure PM:** Evidence of a readily available delirium care pathway in the ED for the documentation of a multicomponent management plan.	○	○	○	○	○	○	○	○	○	○ reset
**18. Process PM:** Proportion of older ED patients with a positive screen for delirium who have a multicomponent management plan documented for the treatment of delirium.	○	○	○	○	○	○	○	○	○	○ reset
**19. Structure PM:** Evidence of local structures available within the ED for older adults with delirium to be placed in a care space with decreased unnecessary stimuli.	○	○	○	○	○	○	○	○	○	○ reset
**20. Process PM:** Proportion of older ED patients with a positive delirium screen who are placed in a care space with decreased unnecessary stimuli.	○	○	○	○	○	○	○	○	○	○ reset

Please Comment:
Use this section to justify your decision (e.g., If you scored between 1-3, why you think a specific PM is not necessary) or if you think something may be missing to better reflect the best practice.

Expand

Category 4: Mangement

BEST PRACTICE #9:

Older ED patients with a positive screen for delirium who are distressed/agitated, or are a risk to themselves or others, are not given antipsychotic medication (e.g., haloperidol) unless de-escalation techniques are ineffective or inappropriate.

Rationale

- The cautious short-term (≤ 7 days) use of haloperidol in specific urgent situations is supported by the evidence
- Haloperidol is still widely used in clinical practice, therefore it is important to articulate parameters of best practice and monitor its use in the ED.

FIRST, INDICATE IF YOU THINK THE BEST PRACTICE IS IMPORTANT & ACTIONABLE.

	Not at all 1	2	3	4	5	6	7	8	Very 9	DK
**Important: Is this important to the care of older adults in the ED?** * must provide value	○	○	○	○	○	○	○	○	○	○ reset
**Actionable: Can this be done in the ED with appropriate resources and tools?** * must provide value	○	○	○	○	○	○	○	○	○	○ reset

Comments:
Use this section to justify your decision (e.g., If you scored between 1-3, why you think the Best Practice is not important or actionable, or if you scored between 7-9 why you think it is very important or actionable).

Expand

SECOND, INDICATE IF YOU THINK THE PERFORMANCE MEASURE (PM) IS NECESSARY.

Necessary: Is the PM necessary to evaluate quality care for older adults in the ED?

21. Process PM:

	Not at all 1	2	3	4	5	6	7	8	Very 9	DK
Proportion of older ED patients with a positive screen for delirium who have been given an antipsychotic medication (e.g., haloperidol) who were documented as being a risk to themselves or others and it is also documented that de-escalation techniques were ineffective or inappropriate.	○	○	○	○	○	○	○	○	○	○ reset

Please Comment:
Use this section to justify your decision (e.g., If you scored between 1-3, why you think a specific PM is not necessary) or if you think something may be missing to better reflect the best practice.

Expand

BEST PRACTICE #10:

Older ED patients with a positive screen for delirium and their family/carers will be given information that explains the condition that meets their needs *(cultural, language, cognitive)*; and family/carers will be encouraged to be present in the ED and involved in care pre and post discharge, *e.g., aiding in cognitive engagement and reorientation of the patient.*

Rationale

- Delirious patients and their family/caregivers may become upset or frightened, and the burden on relatives may decrease when information about the condition is provided.
- Relatives may identify symptoms of delirium quicker than clinicians because of their closeness to the patient.

FIRST, INDICATE IF YOU THINK THE BEST PRACTICE IS IMPORTANT & ACTIONABLE.

	Not at all 1	2	3	4	5	6	7	8	Very 9	DK
**Important: Is this important to the care of older adults in the ED?** * must provide value	○	○	○	○	○	○	○	○	○	○ reset
**Actionable: Can this be done in the ED with appropriate resources and tools?** * must provide value	○	○	○	○	○	○	○	○	○	○ reset

Please Comment:
Use this section to justify your decision (e.g., If you scored between 1-3, why you think the Best Practice is not important or actionable, or if you scored between 7-9 why you think it is very important or actionable).

Expand

SECOND, INDICATE IF YOU THINK EACH PERFORMANCE MEASURE (PM) IS NECESSARY.

Necessary: Is the PM necessary to evaluate quality care for older adults in the ED?

	Not at all 1	2	3	4	5	6	7	8	Very 9	DK
**22. Structure PM:** Evidence of readily available communication tools (e.g., information pamphlets) in the ED to provide older adults with delirium and their family members/caregivers information that explains the condition.	○	○	○	○	○	○	○	○	○	○ reset
**23. Structure PM:** Evidence of information available in English, French, and other languages suited to local demographics (e.g., Indigenous languages) using plain language (e.g., Grade 6 reading level).	○	○	○	○	○	○	○	○	○	○ reset
**24. Process PM:** Proportion of older ED patients with a positive screen for delirium who are given information explaining the condition.	○	○	○	○	○	○	○	○	○	○ reset

Please Comment:
Use this section to justify your decision (e.g., If you scored between 1-3, why you think a specific PM is not necessary) or if you think something may be missing to better reflect the best practice.
