# Supplementary material for: Developing a set of emergency department performance measures to evaluate delirium care quality for older adults: a modified e-Delphi study
Source: BMC Emerg Med. 2024 Feb 15;24:28. doi: 10.1186/s12873-024-00947-6 (PMC10868025; doi:10.1186/s12873-024-00947-6)
Supplement: Supplementary file 2 — Additional file 2: Supplemental File 2. Quality Statement & PM Scores by Delphi Round. [file 12873_2024_947_MOESM2_ESM.pdf]

Supplemental File 2. Quality Statement & PM Scores by Delphi Round

**Quality Statement (QS) or PM**	**Round 1 (N = 22)**<br>**Median**<br>**Scores by tertile (%)**			**Round 2 (n = 21)**<br>**Median**<br>**Scores by tertile (%)**			**Round 3 (N = 22)**<br>**Median**<br>**Scores by tertile (%)**		
	**Low**	**Mid**	**Upper**	**Low**	**Mid**	**Upper**	**Low**	**Mid**	**Upper**
QS 01 Important	8.5			8			8		
	4.55	4.55	90.91	0.00	9.52	90.48	0.00	4.55	95.45
QS 01 Actionable	8			8			8		
	9.09	22.73	68.18	0.00	23.81	76.19	0.00	0.00	100.00
PM 01 Necessary	8			8			8		
	0.00	18.18	81.82	0.00	9.52	90.48	0.00	4.55	95.45
PM02 Necessary	7			7			8		
	9.09	31.82	59.09	4.76	23.81	71.43	0.00	18.18	81.82
PM03 Necessary	8			8			8		
	0.00	22.73	77.27	0.00	14.29	85.71	0.00	9.09	90.91
QS 02 Important	8.5			9			9		
	0.00	9.09	90.91	0.00	4.76	95.24	0.00	0.00	100.00
QS 02 Actionable	8			8			8		
	4.55	27.27	68.18	0.00	19.05	80.95	0.00	9.09	90.91
PM 04 Necessary	8			8			8		
	0.00	9.09	90.91	0.00	4.76	95.24	0.00	4.55	95.45
PM 05 Necessary	8			8			8		
	4.55	13.64	81.82	0.00	14.29	85.71	0.00	13.64	86.36
PM 06 Necessary	7			8			9		
	9.09	22.73	68.18	0.00	28.57	71.43	0.00	27.27	72.73
QS 03 Important	8			9			9		
	0.00	18.18	81.82	0.00	4.76	95.24	0.00	0.00	100.00
QS 03 Actionable	8			9			9		
	0.00	36.36	63.64	0.00	9.52	90.48	0.00	4.55	95.45
PM 07 Necessary	7			8			9		
	0.00	22.73	77.27	0.00	9.52	90.48	0.00	0.00	100.00
PM 08 Necessary	7			8			8.5		
	4.76*	23.81*	71.43*	0.00†	10.00†	90.00†	0.00	0.00	100.00
QS 04 Important	9			9			9		
	0.00	4.55	95.45	0.00	0.00	100.00	0.00	0.00	100.00
QS 04 Actionable	7			8			8		
	13.64	27.27	59.09	9.52	28.57	61.90	4.55	22.73	72.73

PM 09 Necessary	8			9			9		
	9.09	0.00	90.91	0.00	4.76	95.24	0.00	0.00	100.00
PM 10 Necessary	8			8			8		
	0.00	13.64	86.36	0.00	4.76	95.24	0.00	0.00	100.00
PM 11 Necessary	8			8			8		
	13.64	9.09	77.27	4.76	4.76	90.48	0.00	4.55	95.45
QS 05 Important	9			9			9		
	0.00	9.09	90.91	0.00	0.00	100.00	0.00	0.00	100.00
QS 05 Actionable	8			8			8		
	0.00	18.18	81.82	0.00	14.29	85.71	0.00	4.55	95.45
PM 12 Necessary	8			8			8		
	9.09	9.09	81.82	0.00	4.76	95.24	0.00	4.55	95.45
PM 13 Necessary	8			8			8		
	4.55	9.09	86.36	0.00	0.00	100.00	0.00	0.00	100.00
QS 06 Important	7.5			7			8		
	9.09	22.73	68.18	4.76	19.05	76.19	4.55	4.55	90.91
QS 06 Actionable	4			4			4		
	45.45	27.27	27.27	47.62	28.57	23.81	31.82	40.91	27.27
PM 14 Necessary	6			6			6.5		
	18.18	36.36	45.45	14.29	42.86	42.86	9.09	40.91	50.50
QS 07 Important	9			9			9		
	0.00	0.00	100.00	0.00†	0.00†	100.00†	0.00	0.00	100.00
QS 07 Actionable	8			8.5			9		
	4.55	9.09	86.36	5.00†	15.00†	80.00†	0.00	4.55	95.45
PM 15 Necessary	8			8			8		
	0.00*	14.29*	85.71*	0.00	9.52	90.48	0.00	4.55	95.45
PM 16 Necessary	7			7.5			8		
	4.55	22.73	72.73	0.00†	15.00†	85.00†	0.00	0.00	100.00
QS 08 Important	8			8			9		
	0.00	9.09	90.91	0.00†	0.00†	100.00†	0.00	0.00	100.00
QS 08 Actionable**	6			6.5			7		
	31.82	22.73	45.45	25.00†	25.00†	50.00†	9.09	27.27	63.64
PM 17 Necessary	8			8			8		
	0.00*	19.05*	80.95*	0.00†	10.00†	90.00†	0.00	4.55	95.45
PM 18 Necessary	8			8			8		
	0.00	22.73	77.27	0.00†	10.00†	90.00†	0.00	0.00	100.00
PM 19 Necessary	8			8			8		
	4.55	18.18	77.27	0.00	4.76	95.24	0.00	4.55	95.45

PM 20 Necessary	7			7.5			8		
	9.09	22.73	68.18	0.00†	20.00†	80.00†	0.00	13.64	86.36
QS 09 Important	8			9			9		
	0.00	0.00	100.00	0.00†	0.00†	100.00†	0.00	0.00	100.0
QS 09 Actionable	7			7			7		
	9.09	36.36	54.55	5.00†	25.00†	70.00†	0.00	27.27	72.73
PM 21 Necessary	8			8			8		
	4.55	13.64	81.82	5.00†	5.00†	90.00†	0.00	13.64	86.36
QS 10 Important	8.5			9			9		
	0.00	13.64	86.36	0.00†	0.00†	100.00†	0.00	0.00	100.00
QS 10 Actionable	8			8			8		
	4.55	22.73	72.73	0.00†	20.00†	80.00†	0.00	18.18	81.82
PM 22 Necessary	8			8			8.5		
	0.00	13.64	86.36	0.00†	5.00†	95.00†	0.00	0.00	100.00
PM 23 Necessary	8			8			8		
	0.00	9.09	90.91	0.00†	5.00†	95.00†	0.00	0.00	100.00
PM 24 Necessary	7			8			8		
	0.00	36.36	63.64	0.00†	10.00†	95.00†	0.00	4.55	95.45

Note: **, median tertile change and ≥ 15% change in responses between Round 2 and Round 3 (i.e., meaningful change); *, n = 21 (i.e., 1 missing value in Round 1); †, n = 20 (i.e., 1 missing value in Round 2).
